# Supplementary material for: Physalin A, 13,14-Seco-16, 24-Cyclo-Steroid, Inhibits Stemness of Breast Cancer Cells by Regulation of Hedgehog Signaling Pathway and Yes-Associated Protein 1 (YAP1)
Source: Int J Mol Sci. 2021 Aug 13;22(16):8718. doi: 10.3390/ijms22168718 (PMC8395918; doi:10.3390/ijms22168718)
Supplement: Supplementary file 1 [file ijms-22-08718-s001.zip › ijms-1281677-supplementary.pdf]

International Journal of *Molecular Sciences*

MDPI

Supplementary materials

Table S1. Specific Real-time RT-qPCR primer sequences containing.*Nanog, CD44, Oct4, c-myc, Sox2, GLI1, YAP1,* and *β-actin* genes.

Genes	Primers
Nanog	Forward: 5′-ATGCCTCACACGGAGACTGT-3′<br>Reverse: 5′-AAGTGGGTTGTTTGCCTTTG-3′
CD44	Forward: 5′-AGAAGGTGTGGGCAGAAGAA-3′<br>Reverse: 5′-AAATGCACCATTTCCTGAGA-3′
Oct4	Forward: 5′-AGCAAAACCCGGAGGAGT-3′<br>Reverse: 5′-CCACATCGGCCTGTGTATATC-3′
c-myc	Forward: 5′-AATGAAAAGGCCCCCAAGGTAGTTATCC-3′<br>Reverse: 5′-AGCAAAACCCGGAGGAGT-3′
Sox2	Forward: 5′-TTGCTGCCTCTTTAAGACTAGGA-3′<br>Reverse: 5′-CTGGGGCTCAAACTTCTCTC-3′
GLI1	Forward: 5′-CAGGGAAGAGAGCAGACTGAC-3′<br>Reverse: 5′-CAGGAGGATTGTGCTCCA-3′
YAP1	Forward: 5′-GAACCCCAGATGACTTCCTG-3′<br>Reverse: 5′-CTCCTTCCAGTGTTCCAAGG-3′
β-actin	Forward: 5′-TGTTACCAACTGGGACGACA-3′<br>Reverse: 5′-GGGGTGTTGAAGGTCTCAAA-3

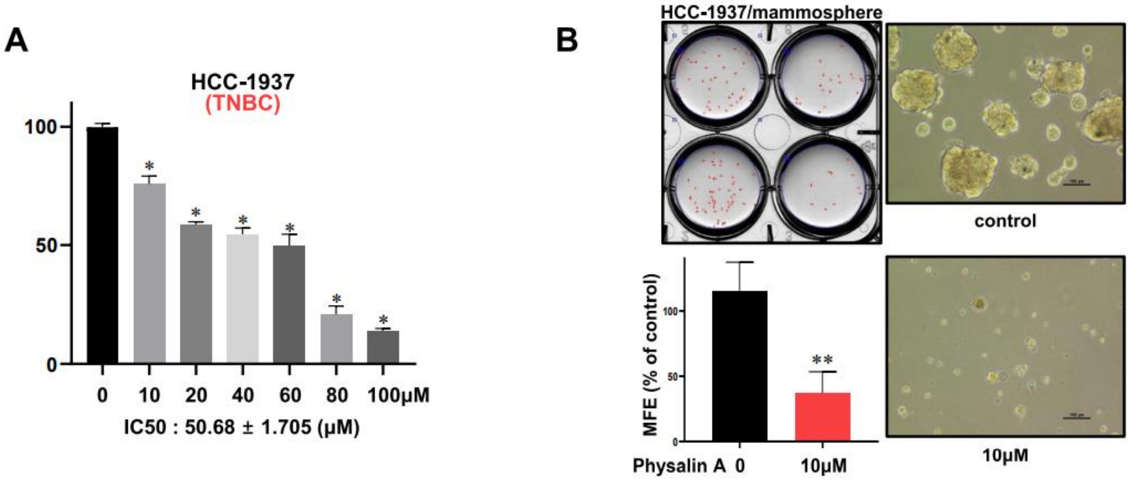

Figure S1. Physalin A inhibits breast cancer cell viability and mammosphere formation efficiency. (**A**) Breast cancer cell line, HCC-1937 (TNBC), was cultured with increasing concentration of physalin A (0, 10, 20, 40, 60, 80, 100 μM) for 24 h. The cytotoxic effect of physalin A was measured using the MTS assay. (**B**) Physalin A inhibits mammosphere-forming ability. HCC-1937 cells (4×10^4 per well) was cultured in 6-well ultralow attachment plates with/without physalin A. Representative mammospheres in the photos were captured by inverted light microscopy (scale bar: 100 μm). The mammosphere formation efficiency (MFE) was determined as shown in the graph. Mean ± SD values from three independent experiments are presented. *$p < 0.05$, **$p < 0.01$.

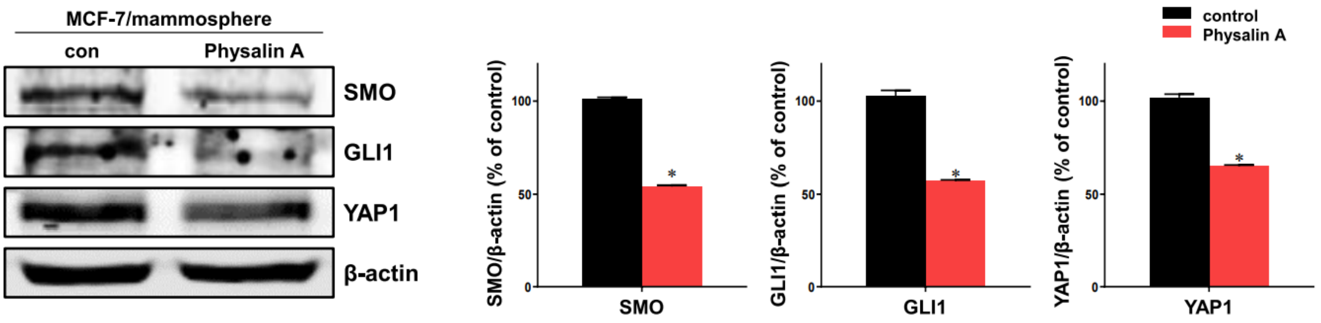

Figure S2. Effect of physalin A on Hedgehog and Hippo signaling pathway of MCF-7 cell line. Physalin A decreased the total protein level of SMO, GLI1, and YAP1 in breast mammospheres. Anti-SMO, anti-GLI1, and anti-GLI1 YAP1 antibody were used for immunoblotting. β-actin was used as the internal control. Western blot images of triplicate experiments are shown as mean ± SD. *$p < 0.05$ vs. DMSO-treated control.
